# Boney Anchylosis Hip-Joint, Operation

**Published:** 1874-02

**Authors:** H. B. Sands


CASE OF BONEY ANCHYLOSIS OF THE HIP-JOINT, SUCCESSFULLY TREATED BY SUBCUTANEOUS DIVISION OF THE NECK OF THE FEMUR.—*By H. B. Sands, M.D.*—The patient, Terence McGrath, a man twenty-five years of age, of fair constitution, but of somewhat irregular habits, suffered from a severe attack of articular rheumatism four years previous to the time of the operation. The disease was most acute in the right hip-joint, which was kept in a flexed position during the patient's convalescence. After the lapse of several months, he was able to leave his bed, but was unable to extend the right thigh, which, until the time when I first saw him, remained perfectly rigid. The thigh was considerably abducted, and was flexed, so as to form an angle of 110° with the vertebral column. The patient could not rest the right foot on the ground without assuming a crouching attitude, and was dependent on the use of crutches in locomotion. No amount of force that I dared to exert caused the slightest movement of the thigh upon the pelvis.

February 12, 1872, I performed subcutaneous division of the neck of the femur, in the following manner: A long, straight, narrow bistoury was thrust through the soft parts just above the great trochanter, and carried directly in front of the cervix femoris, so as to separate the soft parts from this aspect of the bone. The knife was then withdrawn, and a narrow saw an inch and a half in length, and having a long, slender shank, was introduced along the track made by the knife, and the neck of the femur divided. The bone was exceedingly firm, and nearly twenty-five minutes were required to complete the section. Very little blood was lost during the operation; and the external wound, which was hardly three-eighths of an inch in extent, was dressed simply with a piece of lint, a strip of adhesive plaster and a spica-bandage.

After the bone had been severed, it was found necessary to divide the tendons of the adductor longus and the tensor vaginæ femoris. When this had been done, the thigh was immediately and readily extended to a right line with the body. The patient was put to bed, and the limb kept extended by a weight attached to the foot. No inflammation followed the operation; and, on the tenth day, when the dressings were removed, the wound was found to have healed completely, and without suppuration.

He can walk without the assistance of a cane, but his gait is then awkward and unsteady, owing, he says, rather to a weakness of the knee than of the hip. With the aid of a cane, however, he walks quite well, and, for distances not exceeding a mile, without fatigue. The affected limb is three-quarters of an inch shorter than its fellow, and the existence of a false joint is plainly demonstrable.

The question, whether operations of this character may be expected to result in the formation of an artificial joint, is not, in my judgment, a very important one. If the affected limb can be restored to its normal position, and to nearly its normal length, anchylosis will be found, I think, to afford greater security than the best false joint, and to offer no serious obstacle to locomotion, as the movements at the hip-joint are readily compensated for by rotation of the pelvis. In my own case, I suspect that the patient would walk even better than he does at present, if anchylosis had taken place after the deformity had been removed by operation.

